# Enhancing the value of mortality data for health systems: adding Circumstances Of Mortality CATegories (COMCATs) to deaths investigated by verbal autopsy

**DOI:** 10.1080/16549716.2019.1680068

**Published:** 2019-10-25

**Authors:** Laith Hussain-Alkhateeb, Lucia D’Ambruoso, Stephen Tollman, Kathleen Kahn, Maria Van Der Merwe, Rhian Twine, Linus Schiöler, Max Petzold, Peter Byass

**Affiliations:** a School of Public Health and Community Medicine, Sahlgrenska Academy, University of Gothenburg, Gothenburg, Sweden; b Aberdeen Centre for Health Data Science (ACHDS), Institute of Applied Health Sciences, School of Medicine, Medical Sciences and Nutrition, University of Aberdeen, Aberdeen, Scotland, UK; c Department of Epidemiology and Global Health, Umeå University, Umeå, Sweden; d Medical Research Council/Wits University Rural Public Health and Health Transitions Research Unit (Agincourt), School of Public Health, Faculty of Health Sciences, University of the Witwatersrand, Johannesburg, South Africa; e School of Public Health, Faculty of Health Sciences, University of the Witwatersrand, Johannesburg, South Africa; f INDEPTH Network, Accra, Ghana; g Maria van der Merwe Consulting, Nelspruit, South Africa; h Health Metrics, Sahlgrenska Academy, University of Gothenburg, Gothenburg, Sweden; i Stellenbosch Institute for Advanced Study (STIAS), Wallenberg Research Centre at Stellenbosch University, Stellenbosch, South Africa

**Keywords:** Verbal autopsy, health systems, civil registration and vital statistics, social determinants of health, circumstances of death

## Abstract

Half of the world’s deaths and their causes pass unrecorded by routine registration systems, particularly in low- and middle-income countries. Verbal autopsy (VA) collects information on medical signs, symptoms and circumstances from witnesses of a death that is used to assign likely medical causes. To further contextualise information on mortality, understanding underlying determinants, such as logistics, barriers to service utilisation and health systems responses, is important for health planning. Adding systematic methods for categorising circumstantial determinants of death to conventional VA tools is therefore important. In this context, the World Health Organization (WHO) leads the development of international standards for VA, and added questions on the social and health systems circumstances of death in 2012. This paper introduces a pragmatic and scalable approach for assigning relevant Circumstances Of Mortality CATegories (COMCATs) within VA tools, and examines their consistency, reproducibility and plausibility for health policy making, as well as assessing additional effort and cost to the routine VA process. This innovative COMCAT model is integrated with InterVA-5 software (which processes WHO-2016 VA data), for assigning numeric likelihoods to six circumstantial categories for each death. VA data from 4,116 deaths in the Agincourt Health and Socio-Demographic Surveillance System in South Africa from 2012 to 2016 were used to demonstrate proof of principle for COMCATs. Lack of resources to access health care, poor recognition of diseases and inadequate health systems responses ranked highest among COMCATs in the demonstration dataset. COMCATs correlated plausibly with age, sex, causes of death and local knowledge of the demonstration population. The COMCAT approach appears to be plausible, feasible and enhances the functionality of routine VA to account for critical limiting circumstances at and around the time of death. It is a promising tool for evaluating progress towards the Sustainable Development Goals and the roll-out of Universal Health Coverage.

## Background

For as long as there has been interest in systematising data on cause of death – for example, in Graunt’s ‘Bills of Mortality’ for seventeenth century London [1] – highly medical models have dominated thinking on understanding pathways to outcomes. Disease agents (mainly infections), organ systems (particularly for degenerative diseases) and external factors that precede outcomes (such as injuries or treatments) largely underlaid Graunt’s medical cause of death categories. Similar principles underlie modern cause of death classifications, as codified in the World Health Organization’s (WHO) International Classification of Diseases (ICD) system [2]. More recently, ancillary approaches to investigating deaths which include circumstantial factors have been developed, including confidential enquiries (for rare and preventable events) [3] and social autopsy (in the absence of documented circumstances) [4,5]. These methods are highly effective for understanding factors behind individual deaths, but require substantial amounts of work for each case and thus lack feasibility for large-scale population-based work.

Currently, a large proportion of births, deaths and causes of death, particularly in Africa and Asia, are never recorded [6]. Globally, death registration has risen gradually to around 50%. This represents a critical deficiency in national civil registration and vital statistics (CRVS) systems, which aim to register all births and deaths in populations. Functional CRVS systems inform health planning and health services [7], and enable citizens to claim public goods such as housing, welfare, education and justice [8]. However, under-resourced CRVS systems, usually found in poorly resourced countries, often fail to register medical causes, let alone wider circumstances of death [9].

Verbal Autopsy (VA) is a pragmatic approach for assigning causes of death in settings where post-mortem findings and physician certificates are generally unavailable or unreliable. VAs are standardised interviews conducted by trained fieldworkers with final caregivers on the medical signs, symptoms and circumstances surrounding deaths. Interview data are later interpreted into likely causes of death. Interpretation has often been undertaken by physicians, but increasingly automated models are used, which provide more cost-effective, consistent and faster assignment of causes. The WHO curates international VA standards to facilitate cross-national analyses and reporting on levels and causes of otherwise unregistered mortality. WHO-2012 was the first VA standard designed with large-scale automated interpretation in mind [10] and included new indicators on circumstances of death [11], which were retained in the WHO-2016 standard [12]. The InterVA-4 model was developed to process WHO-2012 data [13], updated to InterVA-5 for WHO-2016 [14].

Here we present the concept of Circumstances Of Mortality CATegories (COMCATs), for categorisation of circumstantial determinants of death in parallel with medical causes. This parallel concept does not affect medical cause classifications but provides an additional dimension for understanding deaths (1). COMCATs are not intended to replace confidential enquiry or social autopsy approaches, but enable large-scale assessment of critical limiting circumstances of mortality without significantly burdening the overall VA process. The distribution of COMCATs in a population can track the effectiveness of health services over time and place, thus enabling broader evaluations of interventions encompassing medical, social and health systems aspects of mortality. With the current emphasis from the WHO on achieving Universal Health Coverage (UHC) [15], simple tools that can effectively link health service utilisation to mortality outcomes in populations will be important.

This paper proposes a pragmatic and scalable approach to modelling relevant circumstantial factors (COMCAT) for VA deaths, which could be used on a population-wide basis, adding minimal effort and cost to existing VA processes. The objectives were to (a) ensure consistency and repeatability in assigning circumstantial factors around deaths; (b) achieve comparability over time and place; and (c) demonstrate plausibility. Together, these objectives are intended to assess the utility of the tool for health policy and planning. Here we demonstrate COMCAT as implemented in InterVA-5 on a large population-based dataset from rural South Africa as proof of principle. We do not intend to draw epidemiological conclusions from these demonstration data.

## Methods

### COMCAT design framework

In collaboration with policymakers, health planners and managers at the Mpumalanga Department of Health, South Africa [16], we discussed a potential set of broad social and circumstantial categories into which it would be meaningful to categorise deaths, as a separate but complementary process from assigning medical causes. The six proposed categories, plus a seventh option covering cases where none of the six categories dominates, are shown in Table 1.

**Table 1.** Circumstances Of Mortality CATegories (COMCATs).

COMCATs	Description
Traditions	Traditional practices or beliefs influenced health seeking behaviour and the pathway to death
Emergencies	Sudden, urgent or unexpected conditions leading to death, which probably precluded life-saving actions
Recognition	Lack of recognition or awareness of serious disease (e.g. symptoms or severity) negatively influenced health seeking behaviour
Resources	Inability to mobilise and use resources (e.g. material, transport, financial) hindered access to health care
Health Systems	Problems in getting health care despite accessing health facilities (e.g. related to admissions, treatments and medications)
Inevitability	Death occurred in circumstances that could not reasonably have been averted (e.g. very elderly or recognised terminal conditions)
Multiple	A combination of the above categories affected the pathway to death; no single factor predominated

To enhance the circumstantial power of the overall set of VA indicators, we previously developed a conceptual model relating social and health systems factors to health outcomes. We drew on a classic model of child mortality organising determinants of outcomes as proximate (biological processes and conditions preceding outcomes), intermediate (health systems factors related to care and care processes) and distal (socio-economic and cultural conditions) [17] and more recent models of health systems as ‘core social institutions’, centralising the human and relational nature of the health system [18]. Together with previous empirical work that informed social autopsy methods [4,5], 10 new indicators were developed (Table 2) and piloted in the Agincourt Health and socio-Demographic Surveillance System (HDSS) in Mpumalanga Province, South Africa in 2012, prior to their adoption in the WHO-2012 VA standard [11].

**Table 2.** Questions and substantive responses on social and health systems circumstances of mortality from the WHO-2012 and WHO-2016 WHO VA standards.

WHO-2016 item	Question	Explanation of substantive responses
Id10450	In the final days before death, did she/he travel to a hospital or health facility?	A ‘no’ response indicates no contact with hospital -level services in the days before death (defined as a 24/7 service, but noting in some settings 24/7 facilities may not be called ‘hospitals’).
Id10451	Did she/he use motorised transport to get to the hospital or health facility?	A ‘no’ response indicates that the person who died did not travel to a hospital or health facility by means of motorised transport (car, truck, tractor, motorcycle, scooter or ambulance) during the final illness.
Id10452	Were there any problems during admission to the hospital or health facility?	A ‘yes’ response indicates that the person who died travelled to a hospital or health facility, but then had problems on arrival (delays, paperwork, queues, no staff)
Id10453	Were there any problems with the way she/he was treated (medical treatment, procedures, inter personal attitudes, respect, dignity) in the hospital or health facility?	A ‘yes’ response indicates that the person who died travelled to a hospital or health facility, but then had problems with how they were treated (medical treatment, procedures, inter-personal attitudes, respect, dignity)
Id10454	Were there any problems getting medications, or diagnostic tests in the hospital or health facility?	A ‘yes’ response indicates that the person who died travelled to a hospital or health facility, but then had problems obtaining essential items (drugs, medications or other prescriptions, blood products, and/or diagnostic tests such as lab tests and X-rays, either within the facility or needing to be bought elsewhere).
Id10455	Does it take more than 2 hours to get to the nearest hospital or health facility from the deceased’s household?	A ‘yes’ response indicates that the person who died lived in a household from where it would reasonably take more than 2 hours to reach the nearest 24-hour health facility by the means of transport available to the household members
Id10456	In the final days before death, were there any doubts about whether medical care was needed?	A ‘yes’ response indicates that there were doubts among those assisting in the final illness (family members, etc.) about whether the final illness was sufficiently serious to need treatment at a health facility
Id10457	In the final days before death, was traditional medicine used?	A ‘yes’ response indicates that a major part of treatment for the final illness was provided by any kind of traditional or alternative practitioner (herbal remedies, massages, drinks, foods, amulets, spells or blessings provided by traditional healers, witch doctors or shamans)
Id10458	In the final days before death, did anyone use a telephone or cell phone to call for help?	A ‘no’ response indicates that no telephone of any kind (working landline, or cell phone charged and with credit) was used by those assisting in the final 24 hours of the illness, for example to call for help or arrange transportation
Id10459	Over the course of illness, did the total costs of care and treatment prohibit other household payments?	A ‘yes’ response indicates that the total costs incurred in the final illness were sufficiently great to mean that other kinds of household expenses (food, fuel, travel, education etc.) could not be met, or caused debt or sale of household assets

Many of the conventional VA indicators that are established as part of the interview procedure for determining medical cause of death have implications for or against particular COMCATs. For example, ‘was the baby born in a facility? = yes’ carries a number of implications including the mother’s desire to deliver in a facility, having the agency to get there in time, and enabling health systems options for possible assisted delivery.

As part of the development of the open-source InterVA-5 model [14], a Bayesian probabilistic sub-model was developed to process the indicators from each VA case into likelihoods for the six COMCATs. The conceptual basis and statistical methodology for this was exactly the same as for the established InterVA models [13]. Whereas the cause of death model takes symptoms and processes a database of conditional prior probabilities linking each symptom to each cause, the COMCAT sub-model takes all the indicators from the interview and separately processes conditional probabilities linking responses to each COMCAT. If the likelihood of one of the six COMCATs dominates (i.e. exceeds 50%), that category is assigned to the case; otherwise, the seventh ‘multiple’ category applies. The probability matrix for processing all the VA inputs into the COMCAT outputs was derived on an expert consensus basis as part of the exercise of moving from the InterVA-4 to InterVA-5 model.

### Demonstration population and context

Since 1992, the Agincourt HDSS, run by the MRC/Wits Rural Public Health and Health Transitions Research Unit (Agincourt), has maintained routine surveillance of a population now numbering around 122 500, living in approximately 18,500 households across 31 villages, with one-third being of Mozambican origin [19]. (2) The Agincourt HDSS is amongst Africa’s largest on-going population-based cohorts with routine annual updates documenting information on vital events. Formal sanitation and electricity are limited, but the socio-economic status of the population has improved gradually [20]. One health centre provides 24-h services and satellite clinics provide services during working hours. There are three hospitals 25–40 km from the study area. As well as tracking South Africa’s epidemiological and demographic transitions [21], the Agincourt HDSS site is useful for assessing new approaches for measuring social and health systems determinants at the population level. The Agincourt HDSS is a founding member of the INDEPTH Network [22] and has used the WHO-2012 VA standard since 2012 [10].

### Data analysis

The InterVA-5 model released in 2018 was used to process the complete Agincourt HDSS VA dataset for the period since the WHO-2012 VA standard has been used (2012–2016). InterVA-5 processes WHO-2012 and WHO-2016 standard data [14], both of which include a full set of VA indicators and the 10 circumstantial questions (Table 2) intended to provide additional information about COMCATs [11]. InterVA-5 is the first model that generates COMCAT output as an integral part of VA processing, in parallel to independently generating likely medical causes of death.

The 5-year period covered by the demonstration dataset reflected a period of decreasing HIV/AIDS mortality, following a substantial epidemic that peaked around 2007 [23]. Consequently, the period was characterised by rapidly increasing life expectancy and population ageing as the epidemic declined, and so InterVA-5 outputs for cause-specific mortality and COMCATs are demonstrated as internally age/sex standardised rates. The InterVA-5 model has no inputs related to year of death and so any variations seen over time are inevitably data-driven from the VA interview material.

### Software and licence

The COMCAT concept, integrated with the InterVA-5 model [14], is compatible with openVA software and freely available under www.interva.net, runs in a DOS window on a personal computer; platform independent, and uses FoxPro (compiled into a run-time format) programming language. The software, demonstration material, datasets and code supporting the conclusions of this method are freely available in the GitHub repository https://github.com/peterbyass/InterVA-5

### Ethical clearance

No primary data collection nor specific additional ethical clearance were required for this study. The study data were obtained from the Agincourt HDSS where on-going ethical clearance has been granted by the University of Witwatersrand’s Committee for Research on Human Subjects (Nos. M960720 & M110138). The principle of informed consent was fully respected with the right for refusal or withdrawal from interviews at both individual and household levels. As required by the ethical committee, community consent from civic and traditional leadership was secured at the start of surveillance in 1992 and is reaffirmed annually, as well as obtaining informed verbal consent at individual and household level at each annual follow-up visit.

### Role of the funding sources

The funders had no role in study design, data collection, data analysis, data interpretation, or writing of the report. The corresponding author had full access to all the data in the study and had final responsibility for the decision to submit for publication.

### Findings from applying the COMCAT model

During the 2012–2016 period, 4,116 deaths observed over 464,520 person-years in the Agincourt HDSS were followed up with VA, and InterVA-5 assigned these cases across 54 out of a possible 64 WHO-VA cause of death classifications, including indeterminate. Cause of death findings were also similar to those previously presented for Agincourt HDSS during preceding years using InterVA-4 [23], showing a high HIV-driven burden of infection and increasing non-communicable disease mortality.

By broad categories of cause of death, 36.2% were infections (of which 19.9% was HIV/AIDS and tuberculosis), 10.9% cancer, 14.1% cardiovascular, 11.4% other non-communicable disease (total non-communicable diseases 36.4%), 8.1% injuries, 4.0% pregnancy and neonatal and 15.2% indeterminate. The separately calculated COMCATs were 1.8% traditions, 16.6% emergencies, 17.9% recognition, 20.9% resources, 19.8% health systems, 17.5% inevitability and 5.5% multiple categories. Sex was evenly balanced overall (49.3% of deaths among females) and the only broad cause category with a major sex difference was injury (80.0% among males). Similarly, the only COMCAT with a major sex difference was emergency (62.2% among males).

**Figure 1.**
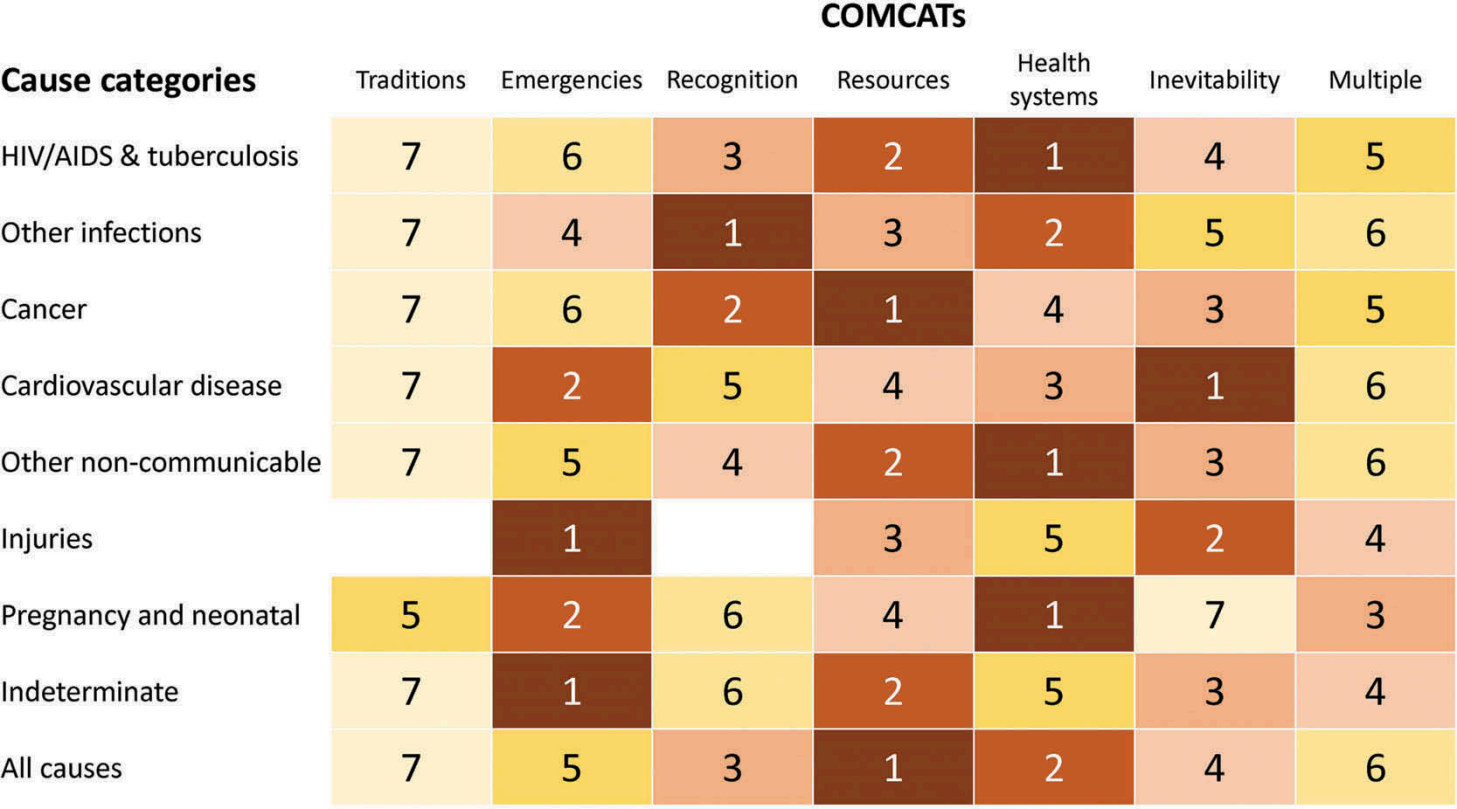
Assigned COMCATs ranked within each major cause of death category, for 4,116 deaths in the Agincourt Health and Demographic Surveillance System.

Figure 1 shows COMCATs ranked for each broad cause category and for all causes. Five of the COMCATs were first-ranked for at least one broad mortality category. The ‘traditions’ COMCAT was not widely assigned in this dataset, and a relatively small proportion had multiple COMCATs rather than one clearly dominating.

**Figure 2.**
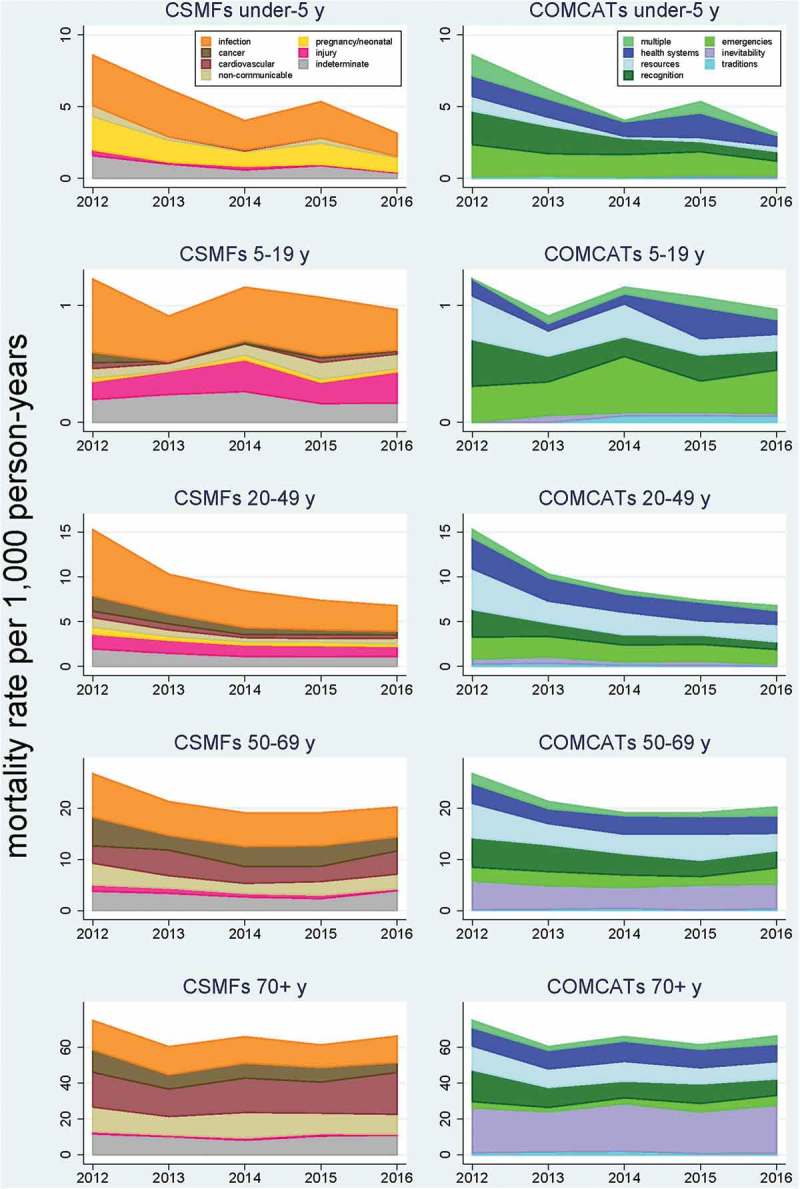
Mortality rates (age-sex standardised) for cause-specific mortality fractions (CSMFs) and Circumstances of Mortality Categories (COMCATs) stratified by year and age groups for 4,116 deaths in the Agincourt Health and Demographic Surveillance System.

Figure 2 shows population-based mortality rates of broad groups of cause of death (given as cause-specific mortality fractions, CSMFs) and their corresponding COMCATs for major age groups (under 5 years, 5–19 years, 20–49 years, 50–69 years and 70-plus years).

## Discussion

The COMCAT concept presented here is an entirely novel approach to extracting an additional dimension of information from routine VA data. While VA methods have mainly been developed and refined to obtain increasingly reliable and cost-effective information on medical causes of death, there is substantial potential value in also considering logistic, social and health system ‘causes’ associated with mortality. While the science of social autopsy has made progress in carrying out in-depth investigations of social factors associated with death, social autopsy is a resource-intensive approach that is unlikely to be routinely applied to millions of deaths or to be part of civil registration and vital statistics systems, despite being a valuable approach for research purposes. Thus, COMCAT is positioned as an alternative means of automatically assigning circumstantial categories to deaths followed up using standard WHO VA protocols, as an added-value output at minimal opportunity cost, and not requiring separate interview processes.

Because of the novelty of the COMCAT concept, there is no comparator against which to evaluate the output in absolute terms. However, the Agincourt HDSS population area has been surveyed in detail over quarter of a century, and is therefore a good starting point in which to assess **plausibility**. The potential value of COMCAT is likely to be more as a practical tool for health managers and planners, rather than merely as a research tool, so it is also useful to assess its **practical utility**. It is useful to consider its relevance in the global context and to make **critical reflections** on the new concept so far.

### Plausibility

From the rankings shown in Figure 1, it is clear that patterns of COMCATs vary considerably across major groups of medical cause of death. This is not surprising, but speaks to the plausibility of the COMCAT modelling, where across a range of examples, consistency was evident between medical causes of death and associated COMCATs. For example, it is entirely likely that injuries would primarily be associated with emergency situations, and secondly with inevitability, in cases where injuries were immediately fatal. Lack of recognition of the potential seriousness of infections is also plausible as a leading COMCAT, given the need to recognise symptoms and obtain treatment promptly. For chronic conditions such as cancers and other non-communicable diseases, resources are likely to be a major constraint, as on-going treatments, medications and travel costs challenge family budgets. Constraints arising from traditional beliefs were not found frequently in this population – although traditional beliefs about health are common in this population, they are often acted upon in parallel with care-seeking from the health services. Furthermore, it may not be the case, although possibly assumed, that traditional therapies are sought at and around the time of death to the extent that they are more generally. Thus, at the top-level, COMCAT ranks seemed plausible in relation to major cause of death groups.

However, plausibility also needs discussing at a more detailed level. Figure 2 shows the same eight major cause of death groups and the seven COMCATs in terms of mortality rates by age group and year. One important reflection here is that InterVA-5 has no sense of time as it processes individual VA records, so variations between years, both in CSMFs and COMCATs, are entirely driven by the VA data presented to the model. The variations over time and demography are very plausible in the context of the decline of the HIV/AIDS epidemic which happened to be underway during the period of observation. This is particularly obvious in the 20–49 years age group, in which total mortality halved over the 5-year period, with much of the change attributable to reductions in deaths related to HIV/AIDS and tuberculosis. Correspondingly for COMCATS, much of the change was reductions in constraints due to recognition and resources, which is entirely consistent with increasing uptake of more easily available HIV and TB treatment as the epidemic progressed.

There was also a substantial reduction in mortality for the under-5 years age group, largely driven by reductions in neonatal and infectious causes of death. This was reflected by reductions in constraints of recognition and resources in COMCATs, while the emergency and health systems COMCATs did not change markedly. This may reflect a continuing lack of access to the 24-h, 7-day health services needed for effective paediatric care. Nevertheless, the analysis also suggests important gains may have occurred in health information and messages being provided to parents as well as a reduction of financial barriers, both of which reflected by reductions in challenges related to recognition and resources, respectively. For the 5–19 years age group, despite the expected much lower overall mortality rates, there was the highest proportion of any age group for injury mortality, and for the COMCATs, the highest proportion of deaths characterised by emergency situations.

Among older people, the 50–69 years age group (who were aged 30–49 years during the early years of the HIV epidemic, when risk of infection was very high) continued later to experience significant but reducing HIV/AIDS-related mortality, while at the same time non-communicable disease was accounting for an increasing proportion of overall mortality, possibly reflecting increasing rates of obesity and other risk factors that are a concern for the future. The major changes in COMCATs in this age group were reductions in recognition and resource constraints. Unsurprisingly the 70-plus years age group had the highest mortality rates, which were nevertheless relatively stable over the 5-year period and dominated by non-communicable diseases. In this age group, COMCATs were dominated by inevitability.

Thus, over all the age groups the COMCATs seem plausibly related to causes of death, in the sense that there are no major issues emerging from the analysis in Figure 2 that appear unlikely from our knowledge of the context and other outputs from the Agincourt HDSS.

### Practical utility

The COMCATs concept is primarily aimed at health service managers and planners who need to be able to monitor population health as comprehensively as possible, using standardised methods. While cause-specific mortality has long been a basic tool for this purpose, there are a number of fundamental drivers and influences which can remain hidden within conventional mortality statistics. For example, a woman who dies after major bleeding around delivery is likely to be represented in cause-specific mortality statistics as a case of obstetric haemorrhage, but that does not differentiate between a woman who dies in a home delivery because she had no means of reaching a facility, compared to a woman who went to a facility but for some reason could not be effectively treated. Differentiating between such scenarios is extremely important for planning and evaluating health services and reducing avoidable mortality.

For managers at provincial/regional or national levels, being able to have basic information such as that shown here in Figure 2, showing current details and trends for the population for which they are responsible, would be invaluable, particularly for encouraging recognition and response to both challenges and successes in service organisation and delivery. From the CSMFs, trends in total mortality and its major cause of death components can be seen very easily. In this demonstration dataset, successful reduction of under-5 mortality and reducing components of mortality associated with HIV/AIDS are clearly evident. At the same time, the proportion of COMCATs due to difficulties with accessing effective health services are not reducing markedly over the 5-year period, which represents a challenge to health authorities. For deaths under 20 years of age, there are also relatively high proportions of the emergency COMCAT. While this may partly reflect risk-related activities among younger people, it is also a matter for concern in this community where free paramedic and ambulance services are not available on a 24-h, 7-day basis. Conversely, if a free emergency service were to be implemented, VA with COMCATs would be a good means of evaluating its effectiveness. Furthermore, records which cannot be assigned a specific medical cause of death (indeterminate), which are always reflected to some extent in any population-based cause of death data, can potentially be elucidated to some extent by their COMCATs.

### Global context

VA occupies an increasingly important place as a tool for understanding and strengthening health systems [24], so adding value to the VA process by incorporating circumstantial classifications for millions of deaths is potentially attractive. Undoubtedly the conventional medical model for conceptualising cause of death will continue to be the central purpose of VA. Despite efforts to use social autopsy jointly with VA to augment the understanding of social and health system barriers to care in some African countries [25,26], these approaches have involved additional work and often been specific to maternal and child health.

The COMCAT system contributes to international calls for strategically reviewing the social determinants of avoidable mortality to better understand health inequalities and social gradients within vital outcomes [27]. The Global Burden of Disease project has postulated a ‘healthcare access and quality’ (HAQ) index as part of their top-down mortality estimation process, but have noted that it is difficult to implement this robustly where mortality inputs depend on conventional VA data [28]. Thus, incorporating individual-level COMCAT assessments into bottom-up VA-based systems may be a helpful strategy.

Against the background of the Sustainable Development Goals (SDGs) and UHC implementation, which will involve major investments in health by many countries, robust indicators for tracking health system effectiveness will be critical. Defining robust indicators however can be difficult. The Director General of the WHO wrote that ‘WHO will develop a measurement system based on Sustainable Development Goal 3.8 indicators to benchmark countries on their attainment of universal health coverage’ [29] COMCAT is an example of a tool generating output indicators that can contribute directly to this process.

### Critical reflections

At this stage, the COMCATs system is at a starting point rather than set in stone. Since the concept of adding circumstantial interpretations of VA data into routine processing is entirely novel, it is certainly not self-evident that the current implementation in InterVA-5 is ideal. It is, however, a functional starting point which appears to add useful information to the overall process of characterising mortality.

In the course of developing COMCATs, we had extensive discussions on the conceptual content of relevant categories and their shorthand labels. Any system of categories is likely to result in a minority of cases overlapping boundaries, which the COMCAT model handles by means of the additional ‘multiple’ COMCAT for instances where the model concludes that no single category predominates. Conceptually this is similar to the inevitable ‘indeterminate’ category in cause of death models. Appropriate labels for COMCATs, even once we were clear about their conceptual content, were difficult to define. We wanted them not to be overly judgemental – but this is hard when the overall objective is characterising circumstances around a death which otherwise might not have happened. Since the InterVA-5 software was released in 2018, we have revised two of the COMCAT labels, from ‘culture’ to ‘traditions’, and from ‘knowledge’ to ‘recognition’, without changing the content of the categories. Nevertheless, further assessment of the tool’s reliability and applicability in other settings is essentially an immediate priority.

InterVA models are committed to following the standard VA interview formats as defined and revised from time to time by WHO. This means we cannot simply invent new VA questions which might be useful for determining COMCATs, although we can propose new items to WHO, which may eventually be included and used, as happened for the 10 circumstantial questions introduced into WHO-2012 and which remain in WHO-2016 (Table 2). Nevertheless, WHO is regularly under pressure from the global community to remove any ‘unnecessary’ items from the VA standards, so all items have to be demonstrably useful.

Since the COMCATs modelling is exclusively based on the WHO VA standard items, using the same mathematical principles as the well-established InterVA approach to modelling medical causes, the COMCAT system has the inherent advantages of not requiring any supplementary interview process, of being totally consistent in interpreting given combinations of VA inputs into COMCATs irrespective of time and place, and therefore not having any significant resource implications.

## Conclusion

This proof-of-principle exercise in associating circumstances of mortality categories with medical causes of death, all implemented within the same VA process, demonstrates the feasibility of adding functionality to VA outputs at very minimal cost and effort. The demonstration dataset from South Africa gave plausible and potentially useful findings for health policy and planning, consistent with what might be expected in that population. Applying the same COMCAT process to data from various settings in the future is likely to provide a basis for comparisons between locations, and longitudinal use of the tool in a particular place should open opportunities to track trends in service provision outcomes. It provides a promising basis for evaluating progress towards the SDGs and measuring outcomes from the roll-out of UHC.

## Paper context

While conceptual frameworks around determinants of pathways to death have long been discussed, tools such as COMCAT for attributing specific circumstantial categories to individual deaths in a systematic, consistent and scalable way have not been widely developed or used. COMCAT, as an adjunct to standard verbal autopsy, is therefore an important step forward for potentially guiding public health via routine feedback to health planners. Monitoring changes over time and space of proportion of deaths determined by health systems issues is among the unfinished global health agenda.
